# Low-normal free thyroxine is associated with a higher prevalence of lower extremity arterial disease in euthyroid type 2 diabetes mellitus

**DOI:** 10.1038/s41598-025-31396-1

**Published:** 2025-12-09

**Authors:** Min Zhang, Chenwen Luo, Jianling Wang, Jieying Wang, Banjun Ruan, Peng Hou, Pu Chen

**Affiliations:** 1 Department of Endocrinology, the First Affiliated Hospital of Xi’an Jiaotong University, Xi’an, 710061 Shannxi P.R. China; 2 Department of Endocrinology, Xi’an No. 9 Hospital, Xi’an, 710054 Shannxi P.R. China; 3 Department of Clinical Medicine, Xi’an Medical College, Xi’an, 710021 Shannxi P.R. China; 4 Department of Public Health, Xi’an Medical College, Xi’an, 710021 Shannxi P.R. China

**Keywords:** Type 2 diabetes mellitus, Lower extremity arterial disease, Thyroid function, Free thyroxine, Endocrine system and metabolic diseases, Diabetes, Thyroid diseases

## Abstract

Recent studies suggest that high-normal concentrations of free triiodothyronine (FT3) were associated with a lower prevalence of microangiopathy in adult euthyroid people with type 1 diabetes. This study was performed to identify the association between thyroid hormones and lower extremity arterial disease (LEAD) in euthyroid patients with type 2 diabetes mellitus (T2DM). A total of 1052 euthyroid T2DM patients were enrolled, including 704 patients with LEAD as observation group and 348 patients with T2DM alone as control group. The differences in clinical characteristics, biochemical indexes, thyroid hormone between the two groups were compared. At the same time, the association between the incidence of LEAD and thyroid hormone was analyzed. The data demonstrated that FT4 levels were significantly lower in the LEAD patients than in the without LEAD patients (16.1 vs. 16.5 pmol/L). The logistic regression analysis revealed that free thyroxine (FT4) was significantly associated with the incidence of LEAD in T2DM patients, and the prevalence of LEAD increased gradually from the highest FT4 quartile to the lowest FT4 quartile (*P* < 0.05). In conclusion, patients with low-normal FT4 had a higher prevalence of diabetic LEAD, suggesting that adjusting FT4 levels may better regulate metabolism and thus reduce lower extremity arterial injury.

## Introduction

Type 2 diabetes mellitus (T2DM) is one of the most serious global health challenges^1^. Over the past two decades, the number of individuals with T2DM has more than doubled^2^. Notably, approximately half (50.1%) of all people living with diabetes are unaware of their condition^3,4^. Patients with T2DM frequently develop lower extremity arterial disease (LEAD). LEAD has been recognized as a key prognostic factor of diabetic foot complications and a major risk factor for amputation^5^. In addition, LEAD has been associated with conventional vascular risk factors such as hyperglycaemia and dyslipidaemia^6,7^.

Thyroid hormone is essential for metabolic regulation, influencing nearly all major biochemical processes related to energy distribution, such as glucose processing, gluconeogenesis, adipogenesis, lipolysis, and insulin introduction. In metabolically abnormal T2DM patients, normal thyroid hormone levels are linked to better metabolic regulation, which may help reduce the occurrence of diabetic complications such as LEAD. Growing evidence suggests that even within the clinically normal reference range, variations in thyroid hormone levels may have significant pathophysiological consequences. In euthyroid patients with type 1 diabetes, high-normal concentrations of free triiodothyronine (FT3) are associated with a lower incidence of microvascular complications and improved metabolic control^8^.

Thus, the association between thyroid hormones and LEAD in euthyroid patients with T2DM was evaluated in this study. Totally, 1052 T2DM patients with normal thyroid function were enrolled, including 704 patients with LEAD as observation group and 348 patients with T2DM alone as control group. The differences in clinical characteristics, biochemical indexes, thyroid hormone between the two groups were compared. At the same time, the association between the incidence of LEAD and thyroid hormone was analyzed. The data suggested that patients with low-normal free thyroxine (FT4) had a higher prevalence of diabetic LEAD.

## Materials and methods

### Patients

This study was approved by the Ethics Committee of Xi’an Jiaotong University Health Science Center (No. XJTU1AF2021LSK-051). From January 2014 to May 2020, patients with initial diagnosis of T2DM from the First Affiliated Hospital of Xi’an Jiaotong University were enrolled into the present study. T2DM was diagnosed based on the World Health Organization (WHO) diagnostic criteria^9^, which includes: diabetic symptoms and random blood glucose ≥ 11.1mmol/L, or fasting blood glucose ≥ 7.0mmol/L (no calorie intake for at least 8 h), or blood glucose ≥ 11.1mmol/L at 2 h of an oral glucose tolerance test (OGTT). Diabetic patients with LEAD diagnostic criteria were as follows (meet any of the following conditions): (1) Patient’s resting ankle brachial index (ABI) ≤ 0.90, regardless of whether the patient has symptoms of lower limb discomfort. (2) Patients with lower extremity discomfort during exercise and resting ABI ≥ 0.90. For example, ABI decreased by 15% −20% after treadmill flat test. (3) Patients should be diagnosed with severe limb ischemia (CLI) if the resting ABI is < 0.40 or the ankle artery pressure is < 50 mmHg or the toe artery pressure is < 30 mmHg. Exclusion criteria were as follows: (1) Participants had acute complications of diabetes or other chronic complications. (2) There is hypothalamus or pituitary diseases, the thyroid dysfunction. (3) Infectious diseases, abnormal blood pressure and patients with tumors. (4) Pregnant women and lactating women were excluded. Eventually, 1052 patients were included in this research. Among them, 704 (66.92%) had T2DM with LEAD and 348 (33.08%) had T2DM without LEAD.

### Clinical and laboratory examination

The basic information of all patients was inquired and recorded by an endocrinology department professional nurse on the day when the patient entered the hospital. After fasting for 8 h, 44 biochemical indexes were detected by intravenous blood sampling in the morning. The thyroid metabolic indexes were measured by radioimmunoassay (Beijing North Institute of Biotechnology, China), and the indexes were: FT4, tetraiodothyronine (T4), FT3, triiodothyronine (T3), thyroid stimulating hormone (TSH) and thyroid peroxidase antibody (TPOAb). Other biochemical indexes were detected by clinical automatic biochemical analysis system (Hitachi LABOSPECT 008, Japan), including total protein (TP), albumin (ALB), prealbumin(PA), globulin (GLB), aminotransferase (ALT), aminotransferase (AST), indirect bilirubin (I-BIL), direct bilirubin (DBIL), total bilirubin (TBIL), total bile acid (TBA), alkaline phosphatase (ALP), lactate dehydrogenase (LDH), creatine kinase (CK), creatine phosphokinase-MB (CK-MB), α-hydroxybutyrate dehydrogenase (α-HBDH), superoxide dismutase Carbon dioxide (SOD), low-density lipoprotein (LDL), carbon dioxide combining power (CO2CP), triglycerides (TG), high-density lipoprotein (HDL), creatinine (Cr), anion gap (AG), calcium (Ca), potassium (K), phosphorus (P), chlorine (CL), magnesium (Mg), sodium (Na), blood urea nitrogen (BUN), uric acid (UA), glucose (GLU), retinol binding protein (RBP), glycated albumin (GA), apolipoprotein A (Apo A), apoprotein B (Apo B), apoprotein E (Apo E), lipoprotein a (Lp-a), total cholesterol (TC) and cystatin C (Cys C).

### Statistical analysis

SPSS 22.0 statistical software was used for data analysis in this study. Categorical variables were expressed as percentages, and the continuous variables that are not normally distributed were represented by quartiles. The differences between the two groups of continuous variables were tested by Mann-Whitney U test, and the classification variables were tested by the Chi-square test. The association between FT4 and other clinical indicators in LEAD group was analyzed by Spearman correlation analysis. FT4, T4, FT3 and T3 were divided into four levels according to their respective quartiles, and TSH was divided into two levels. Then to compare the prevalence of LEAD between each group by the Chi-square test. The logistic regression analysis was used to analyze the association between thyroid hormone levels and the prevalence of LEAD. *P* value < 0.05 was considered statistically significant.

## Results

### Clinical characteristics of the participants in this study

The clinical data and biochemical test items of the participants were shown in Table 1. The LEAD group, including 704 T2DM patients with LEAD, the age quartile was 56 (49–63) years and 71.2% were male. For the control group, 348 T2DM patients without LEAD were included, the age quartile was 52 (42–62) years and 67.8% were male. Compared with the control group, the LEAD group patients had lower levels of biochemical indicators TP, ALB, ALT, AST, LDH, α-HBDH, SOD, AG, Ca, K, P, Mg, RBP, and Apo E. In terms of thyroid hormones, FT4 levels were significantly lower in the LEAD patients than in the without LEAD patients. However, there were no significant differences in FT3, T3, T4 and TSH levels between the two groups.

**Table 1:** Clinical characteristics of T2DM patients with or without LEAD.

Characteristics	Total(1052)	Without-LEAD(348)	LEAD(704)	*P*
Sex(male)	737(70.1%)	236(67.8%)	501(71.2%)	0.265
Age	55(47–63)	52(42–62)	56(49–63)	0.000***
FT3	4.83(4.23–5.46)	4.91(4.25–5.52)	4.8(4.22–5.43)	0.068
FT4	16.2(14.1–18.3)	16.5(14.3–18.8)	16.1(14–18)	0.005**
T3	1.19(1.02–1.37)	1.18(1.02–1.37)	1.20(1.02–1.37)	0.832
T4	7.37(6.25–8.73)	7.50(6.41–8.80)	7.34(6.21–8.73)	0.331
TSH	1.75(1.1–2.54)	1.65(1.06–2.47)	1.80(1.11–2.56)	0.218
Positive TPOAb	140(13.3%)	53(15.2%)	87(12.4%)	0.197
TP	66.6(63.2–70.68.2.68)	67.2(63.43–71.68)	66.5(63–70.2.2)	0.012*
ALB	41.2(38.8–43.9)	42.05(39–44.7.7)	40.9(38.7–43.7)	0.001**
PA	255.9(219.2–292.1.2.1)	257.3(220.9–294.9.9.9)	254.85(218.5–291.68.5.68)	0.787
GLB	25.3(23.13–27.68)	25.25(23.23–28.23)	25.3(23.1–27.6)	0.382
ALT	20(14–31)	22(15.25–37.25)	20(14–29.75.75)	0.023*
AST	19(16–24)	19.5(16–26)	18(15–23)	0.006**
I-BIL	8.1(6.4–10.7)	8.2(6.6–10.68.6.68)	8(6.3–10.7)	0.415
DBIL	3.5(2.6–4.6)	3.6(2.7–4.7)	3.4(2.6–4.6)	0.178
TBIL	11.5(9.1–15.3)	11.6(9.23–15.2)	11.5(8.9–15.38.9.38)	0.375
TBA	3.7(2.5–5.8)	3.7(2.5–5.6)	3.75(2.5–5.98)	0.333
ALP	67.5(55–83)	69(55–86)	67(55.25–82.25)	0.235
LDH	179(159–201)	184(162–206)	177(158–197)	0.006**
CK	74(56–105)	74(55–106.75.75)	74.5(56–103.75.75)	0.939
CK-MB	13(11–16)	12(10–16)	13(11–16)	0.036*
α-HBDH	144(127–162)	148(132–168)	142(126–159)	0.000***
SOD	173.05(158.3–189.55.3.55)	176.95(160.5–194.6.5.6)	171.5(156.93–186.98.93.98)	0.003**
LDL	2.44(1.92–3.01)	2.42(1.87–3.06)	2.46(1.95–3.0.95.0)	0.649
CO2CP	22.2(20–24.6.6)	22(19.83–24.08)	22.4(20.1–24.9)	0.067
TG	1.44(0.99–2.2)	1.49(1.0–2.29.0.29)	1.42(0.98–2.16)	0.356
HDL	0.98(0.85–1.15)	0.98(0.84–1.19)	0.97(0.85–1.14)	0.476
Cr	59(50–67.75.75)	59(50–68)	58(50–67)	0.487
AG	23.5(20.7–26.5)	23.85(21.5–26.6)	23.2(20.33–26.5)	0.006**
Ca	2.21(2.14–2.29)	2.24(2.16–2.31)	2.2(2.13–2.28)	0.000***
K	3.89(3.66–4.15)	3.95(3.73–4.19)	3.85(3.62–4.13)	0.000***
P	1.13(1.02–1.25)	1.15(1.02–1.28)	1.12(1.02–1.23)	0.013*
CL	103.5(101.3–105.78.3.78)	103.4(101.13–105.7)	103.6(101.3–105.8.3.8)	0.393
Mg	0.89(0.85–0.95)	0.9(0.85–0.96)	0.89(0.84–0.94)	0.012*
Na	142(141–144)	142(141–144)	142(141–144)	0.516
BUN	5.37(4.52–6.35)	5.25(4.46–6.24)	5.43(4.59–6.43)	0.218
UA	316(266.25–377.75.25.75)	327.5(267–382.5.5)	312.5(266–372)	0.232
GLU	6.8(5.56–8.73)	6.69(5.56–8.67)	6.84(5.56–8.83)	0.724
RBP	51.1(38.33–64.6)	55.5(40.63–67.58)	49.25(37.33–61.88)	0.000***
GA	18.8(15.6–23.48.6.48)	18.8(15.63–22.8)	18.8(15.6–24)	0.516
Apo A	1.18(1.05–1.30)	1.19(1.06–1.33)	1.17(1.05–1.29)	0.057
Apo B	0.82(0.067–0.97.067.97)	0.8(0.65–0.98)	0.82(0.68–0.96)	0.753
Apo E	34.8(27.5–45.1)	36.8(29.23–48.78)	34.1(27.13–43.2)	0.001**
Lp(A)	117(59–227)	129(61.25–229)	114.5(57.25–225)	0.197
TC	4.12(3.47–4.76)	4.14(3.44–4.86)	4.11(3.47–4.70)	0.525
CysC	0.82(0.73–0.95)	0.82(0.72–0.94)	0.82(0.73–0.95)	0.850

Normal reference range: TSH 0.25–5.0.25.0 µIU/mL, FT4 9.05–25.5 pmol/L, FT3 2.91–9.08 pmol/L, T4 4.2–13.5 µg/dL, T3 0.78–2.2 ng/mL. *P < 0.05;**P < 0.01;***P < 0.001.

### Relationship of FT4 and clinical characteristics in LEAD group

As shown in Table 2, spearman correlation analysis was used to analyze the association between clinical indicators and FT4 in the T2DM patients with LEAD. The results showed that FT4 level was significantly positively correlated with TP, ALB, PA, I-BIL, TBIL, α-HBDH, SOD, HDL, AG, Ca, RBP, and Apo A. At the same time, FT4 level was significantly negatively correlated with age, TBA, ALP, CO2CP and GA.

**Table 2:** Association between FT4 and clinical characteristics among T2DM subject with LEAD.

		Sex	Age	TP	ALB		PA		GLB	ALT	AST	I-BIL
r value		0.030	−0.078	0.163	0.243		0.125		0.002	−0.051	−0.017	0.077
*P*		0.423	0.039	0.000	0.000		0.001		0.958	0.173	0.660	0.042
		DBIL	TBIL	TBA	ALP		LDH		CK	CK-MB	α-HBDH	SOD
r value		0.068	0.08	−0.082	−0.094		0.017		−0.018	−0.031	0.105	0.23
*P*		0.072	0.033	0.029	0.013		0.657		0.636	0.418	0.006	0.000
		LDL	CO2CP	TG	HDL		Cr		AG	Ca	K	P
r value		0.035	−0.079	−0.01	0.124		−0.030		0.102	0.15	−0.001	−0.006
*P*		0.355	0.036	0.786	0.001		0.430		0.007	0.000	0.976	0.871
		Cl	Mg	Na	BUN		UA		GLU	RBP	GA	Apo A
r value		−0.063	0.014	0.009	0.009		0.046		−0.011	0.221	−0.074	0.16
*P*		0.095	0.718	0.816	0.818		0.218		0.772	0.000	0.048	0.000
		Apo B	Apo E	Lp(A)	TC		Cys C					
r value		0.039	0.026	0.010	0.028		−0.069					
*P*		0.301	0.499	0.794	0.456		0.067					

Association between FT4 and clinical characteristics were tested by Spearman correlation analysis. *P* value < 0.05 was considered statistically significant.

### The prevalence of LEAD among FT4, T4, FT3, T3, and TSH groups

As shown in Table 3, the prevalence of LEAD in FT4 quartiles was 70.88% (< 14.1 pmol/L), 68.65% (14.1–16.2 pmol/L), 69.09% (16.2–18.3 pmol/L), and 59.09% (≥ 18.3 pmol/L), respectively. Compared with the last quartile group, we found that when the level of FT4 was lower than 18.3 pmol/L, the prevalence of LEAD remarkably increased (*P* < 0.05). There were no significant differences among T4 groups, FT3 groups, T4 groups, and TSH groups in the prevalence of LEAD.

**Table 3:** The prevalence of LEAD among FT4, T4, FT3, T3, and TSH groups.

Group	Total Number	LEAD		X^2^	*P*
		Number	Prevalence (%)		
TSH <2.5	776	514	66.24	0.623	0.430
≥ 2.5	276	190	68.84		
FT4 <14.1	261	185	70.88	10.086	0.018*
14.1–16.2	252	173	68.65		
16.2–18.3	275	190	69.09		
≥ 18.3	264	156	59.09		
FT3 <4.23	259	176	67.95	5.173	0.160
4.23–4.83	263	189	71.86		
4.83–5.46	267	172	64.42		
≥ 5.56	263	167	63.50		
T4 <6.25	262	188	71.76	4.089	0.252
6.25–7.37	263	168	63.88		
7.37–8.73	262	172	65.65		
≥ 8.73	265	176	66.42		
T3 <1.02	253	169	66.80	41.278	0.734
1.02–1.19	268	174	64.93		
1.19–1.37	259	180	69.50		
≥ 1.37	272	181	66.54		

The prevalence of LEAD among FT4, T4, FT3, T3, and TSH groups were tested by Chi-square test. *P < 0.05.

### Association between thyroid hormone levels and the prevalence of LEAD

Logistic regression analysis was used to analyze the association between FT4, T4, FT3, T3 and TSH and the prevalence of LEAD, and the results were shown in Table 4. The last group FT4 Q4 (≥ 18.3 pmol/L) was used as a reference for comparison, the crude ORs (95% CI) for LEAD in FT4 Q1 (< 14.1 pmol/L), FT4 Q2 (14.1–16.2 pmol/L), and FT4 Q3 (16.2–18.3 pmol/L) were 1.685 (1.173–2.422), 1.516 (1.056–2.177), and 0.548 (1.086–2.206) (*P* = 0.005, *P* = 0.024, *P* = 0.016)(Model 1), respectively. After adjusting for age, sex, TP, ALB, ALT, AST, LDH, CK-MB, α-HBDH, SOD, AG, Ca, K, P, Mg, RBP and Apo E, the ORs (95% CI) for LEAD in FT4 Q1 (< 14.1 pmol/L), FT4 Q2 (14.1–16.2 pmol/L), and FT4 Q3 (16.2–18.3 pmol/L) were 1.415 (0.940–2.132), 1.393 (0.942–2.059), and 1.569 (1.071–2.297) (*P* = 0.097, *P* = 0.097, *P* = 0.021) (Model 2), respectively. TSH, FT3, T4 and T3 groups had no significant association with LEAD.

**Table 4:** Logistic regression analysis of thyroid hormone levels with LEAD.

Characteristics	Groups	Number	Model 1 OR (95% CI)	*P*	Model 2 OR (95% CI)	*P*
TSH	< 2.5 ≥ 2.5	776 276	0.888(0.661–1.193) Reference	0.430	0.943(0.685–1.299) Reference	0.721
FT4	< 14.1 14.1–16.2 16.2–18.3 ≥ 18.3	261 252 275 264	1.685(1.173–2.422) 1.516(1.056–2.177) 1.548(1.086–2.206) Reference	0.005** 0.024* 0.016*	1.415(0.940–2.132) 1.393(0.942–2.059) 1.569(1.071–2.297) Reference	0.097 0.097 0.021*
FT3	< 4.23 4.23–4.83 4.83–5.46 ≥ 5.56	259 263 267 263	1.219(0.849–1.751) 1.468(1.016–2.121) 1.041(0.730–1.484) Reference	0.284 0.041* 0.825	0.854(0.558–1.308) 1.194(0.797–1.789) 0.945(0.646–1.382) Reference	0.469 0.390 0.772
T4	< 6.25 6.25–7.37 7.37–8.73 ≥ 8.73	262 263 262 265	1.285(0.887–1.861) 0.894(0.625–1.279) 0.966(0.674–1.3866) Reference	0.185 0.541 0.853	1.175(0.783–1.764) 0.757(0.513–1.118) 0.932(0.634–1.369) Reference	0.436 0.162 0.718
T3	< 1.02 1.02–1.19 1.19–1.37 ≥ 1.37	253 268 259 272	1.012(0.703–1.454) 0.931(0.652–1.328) 1.146(0.795–1.651) Reference	0.925 0.692 0.466	0.751(0.493–1.142) 0.722(0.488–1.069) 1.157(0.783–1.709) Reference	0.181 0.104 0.464

Model 1: crude model. Model 2: adjusted for age, sex, TP, ALB, ALT, AST, LDH, CK-MB, α-HBDH, SOD, AG, Ca, K, P, Mg, RBP and Apo E. The logistic regression analysis was used to analyze the association between thyroid hormone levels and the prevalence of LEAD. *P value < 0.05; **P < 0.01.

## Discussion

Thyroid hormones serve as key regulators of energy balance and lipid metabolism^10^. However, abnormal levels of these hormones can disrupt energy expenditure, cholesterol homeostasis, lipid catabolism, and gluconeogenesis^11^. Beyond metabolic effects, thyroid dysfunction is implicated in the pathogenesis of various diseases. Results from a prospective cohort study from the International Cooperation for Thyroid Research show that subclinical hypothyroidism (SCH) is associated with increased coronary heart disease mortality^12^. Meanwhile, another study has shown that in normal thyroid patients with type 1 diabetes, high-normal FT3 levels are associated with a lower incidence of microangiopathic complications and better metabolic control^8^. In line with these findings, these results showed that a low-normal FT4 level is a significant risk factor for the development of LEAD in euthyroid patients with T2DM.

LEAD is a chronic ischemic disease of lower extremities and a frequent complication of diabetes. Key risk factors include smoking, diabetes, hypertension, and hypercholesterolemia^13^. Thyroid hormone receptors are present in the myocardium and vascular tissue, and subtle variations in thyroid hormone concentrations can affect cardiovascular physiology^14^. Some studies have reported that thyroid hormones affect the heart and vasculature, with mild hypothyroidism promoting atherosclerosis, likely through impaired vascular endothelial function^15^. Adem Gungor et al. found that FT4 was significantly associated with endothelial injury in overt hypothyroidism^16^. Consistent with these findings, our data revealed that FT4 levels were lower in patients with LEAD and the prevalence of LEAD increased as the quartile of FT4 levels decreased. Therefore, our results suggested that even within the euthyroid range, low-normal FT4 may cause LEAD in T2DM patients by directly affecting the vascular endothelial system.

At the same time, thyroid hormone plays a vital role in maintaining systemic physiological functions and significantly regulates lipid metabolism. Dyslipidemia, in turn, contributes to the development of microvascular complications. Thyroid hormone deficiency reduces the activity of lipoprotein lipase, sterol regulatory element binding protein-2, and apolipoprotein A1 (Apo A1)^17^, and is associated with elevated higher levels of atherosclerotic lipoprotein^18^. The present study found FT4 was positively correlated with HDL and Apo A. Low-normal levels of FT4 may promote abnormal lipid metabolism in patients, leading to atherosclerosis and peripheral vascular damage.

T2DM is characterized as a long-term, chronic low-grade inflammatory state, with various immune and inflammatory mediators contributing to both the pathogenesis of the disease and its complications^19^. In T2DM patients with LEAD, elevated levels of acute-phase proteins and cytokines correlate with ulcer size and infection severity^20^. Similarly, hypothyroidism has also been related with increased inflammatory markers such as C-reactive protein (CRP)^21^. Therefore, inflammation may serve as a common pathway linking T2DM and hypothyroidism, and could play a role in the relationship between abnormal thyroid hormones and LEAD.

Low-normal FT4 levels may serve as a risk factor for LEAD in the patients with T2DM, potentially contributing to vascular endothelium directly through the thyroid hormone receptor of vascular endothelium, as well as indirectly through lipid metabolic abnormalities. Levothyroxine supplementation has been shown to ameliorate atherosclerosis and endothelial dysfunction in patients with subclinical hypothyroidism^22–24^. Therefore, we can monitor the thyroid hormone level of patients, and give low dose of thyroxine when necessary to maintain the higher level of FT4 level in the normal range, so as to reduce the occurrence and development of LEAD in T2DM patients.

This study has several limitations. There was a discrepancy in the sample sizes between the observation and control groups. The cross-sectional design prevents causal inference, and the lack of comprehensive autoimmune markers limits the interpretation of the role of autoimmunity and inflammation. Potential residual confounding should also be considered.

In conclusion, patients with low-normal FT4 had a higher prevalence of diabetic LEAD, suggesting that adjusting FT4 levels may better regulate metabolism and thus reduce lower extremity arterial injury.

## Data Availability

All data generated or analyzed during this study are included in this article. Further inquiries can be directed to the corresponding author.
